# Standardisierte histomorphologische Aufarbeitung von Peritonealbiopsien im Rahmen des Deutschen Peritonealbiopsieregisters (GRIP, German Registry In PD)

**DOI:** 10.1007/s00292-020-00815-7

**Published:** 2020-09-07

**Authors:** Frederick Pfister, Maike Büttner-Herold, Benno Kitsche, Dirk R. Bulian, Jan Kielstein, Reinhard Wanninger, Gabriele Eden, Dominik Alscher, Michael Nebel, Vedat Schwenger, Kerstin Amann

**Affiliations:** 1 Abt. Nephropathologie, Pathologisches Institut, Universitätsklinikum Erlangen, Krankenhausstr. 8–10, 91054 Erlangen, Deutschland; 2 Kuratorium für Dialyse und Nierentransplantation e. V. (KfH) Neu-Isenburg, Neu-Isenburg, Deutschland; 3 Klinik für Viszeral‑, Tumor-, Transplantations- und Gefäßchirurgie, Lehrstuhl Chirurgie I, Klinikum der Universität Witten/Herdecke, Köln-Merheim, Deutschland; 4 Klinik für Nieren- und Hochdruckkrankheiten, Medizinische Klinik V, Städt. Klinikum Braunschweig, Braunschweig, Deutschland; 5 Robert-Bosch-Krankenhaus, Stuttgart, Deutschland; 6 Klinik für Nieren‑, Hochdruck- und Autoimmunerkrankungen, Transplantationszentrum Stuttgart, Katharinenhospital Stuttgart, Klinikum Stuttgart, Stuttgart, Deutschland

**Keywords:** Peritoneum, Peritonealdialyse, Morphologie, Fibrose, EPS, Peritoneum, Peritoneal dialysis, Morphology, Fibrosis, EPS

## Abstract

Das Peritoneum stellt als seröse Haut, die mit ihrem viszeralen und parietalen Anteil den Bauchraum auskleidet, ein interessantes Organ dar, welches bei der sog. Peritoneal- oder Bauchfelldialyse (PD) klinische Beachtung findet. Bei diesem Nierenersatzverfahren wird die Semipermeabilität des Peritoneums genutzt, um mittels unterschiedlich osmolarer Dialyseflüssigkeiten die sog. harnpflichtigen Substanzen aus dem Körper zu eliminieren. Dies ist insbesondere bei jungen Patienten eine ideales Nierenersatzverfahren und funktioniert in der Regel zumindest einige Zeit sehr gut. Vorschäden des Peritoneums durch die Grunderkrankung der chronischen Niereninsuffizienz oder assoziierte Komorbiditäten sowie v. a. entzündliche Veränderungen während der PD führen zu einem morphologischen Umbau des Peritoneums mit der Konsequenz des Verlusts der Filtereigenschaften, sodass die PD beendet und auf ein anderes Nierenersatzverfahren gewechselt werden muss. Die Kenntnis des morphologischen Umbaus des Peritoneums sowie möglicher begünstigender Faktoren, zu denen es derzeit noch zu wenige Daten gibt, ist wichtig für die Therapie und Prognose der Patienten, die mit PD behandelt werden. Aus diesem Grund wurde vor einigen Jahren das Deutsche Peritonealbiopsieregister (GRIP, German Registry In PD) gegründet, das mittlerweile knapp 1700 Biopsate umfasst und an diesen standardisiert klinische und histomorphologische Parameter erhebt und dokumentiert.

## Abstract (en)

The peritoneal lining of the abdominal cavity consists of a parietal and visceral sheet. The serosa is an interesting organ, which in medical practice is particularly important in the context of chronic peritoneal dialysis (PD). This method of renal replacement therapy utilizes the semipermeability of the peritoneal surface by applying PD solutions of differing osmolarity to eliminate toxic substances and regulate fluid and electrolyte equilibrium. This method is an ideal approach especially for younger patients and is very effective at least for some time. Pre-existing injury to the peritoneum, for example as a consequence of chronic renal insufficiency or associated comorbidities and inflammatory changes that develop during PD, results in a structural remodelling of the serosa. As a consequence, the filtering function of the serosa is lost and PD has to be replaced by another renal replacement therapy. Thorough knowledge of the morphology of peritoneal changes as well as of the risk factors is of paramount importance for therapeutic management and prognosis of PD patients. In order to take this into account, the German Registry In Peritoneal Dialysis (Deutsches Peritonealbiopsieregister, GRIP) was founded a few years ago, which now includes roughly 1700 biopsies, of which detailed clinical and histomorphological information was systematically acquired and collected.

Die Bauchfelldialyse (Peritonealdialyse, PD) ist ein Nierenersatzverfahren, welches im Vergleich zur Hämodialyse mit vielen Vorteilen assoziiert ist und sich v. a. für jüngere Patienten*innen eignet. Darüber hinaus bietet sie sich auch bei spezifischen Komorbiditäten, wie z. B. dem kardiorenalen Syndrom, bei älteren Patient*innen an [6]. Sind mehrere Jahrzehnte mit einer Nierenersatztherapie zu überbrücken, so eignet sich die PD zur Einleitung eines Nierenersatzverfahrens.

Voraussetzung für eine erfolgreiche und kontinuierliche PD ist die Integrität und Funktion des Peritoneums, da dieses bei der PD als „Dialysemembran“ eingesetzt wird. Es ist bekannt, dass das Peritoneum bereits im Verlauf einer chronischen Niereninsuffizienz, d. h. schon vor Beginn der eigentlichen Dialyse, strukturelle Veränderungen aufweist und daher die Dialysecharakteristika (i.S. von Ultrafiltration und Entgiftung) bereits zu Beginn der PD bei einigen Patienten eingeschränkt sein können. Weiterhin ist bekannt, dass das Peritoneum im Verlauf der PD verschiedenen Noxen ausgesetzt ist. Die morphologische Veränderung des Peritoneums ist in erster Linie durch eine Toxizität der Dialyseflüssigkeiten bedingt, geht aber auch auf verschiedenen Komorbiditäten und Stoffwechselerkrankungen, wie z. B. Diabetes mellitus, sowie v. a. entzündliche Veränderungen zurück. Die Kenntnis der Ausgangsbedingungen des Peritoneums zu Beginn der PD sowie die im Folgenden eintretenden morphologischen Veränderungen und ihre funktionellen Konsequenzen sind für die Behandlung jedes einzelnen PD-Patienten aber auch für die Optimierung dieses Nierenersatzverfahrens von großer klinischer Bedeutung.

Aus diesem Grund wurde vor einigen Jahren das Deutsche Peritonealbiopsieregister (*GRIP, G*erman *R*egistry *I*n *P*D) ins Leben gerufen, das von der Kommission Peritonealdialyse und Heimdialyse der Deutschen Gesellschaft für Nephrologie (DGfN) geleitet und von der DGfN, dem Kuratorium für Dialyse und Nierentransplantation e. V. (KfH) und der Patienten-Heimversorgung gemeinnützige Stiftung (PHV) finanziell und ideell unterstützt wurde. An logistischen Voraussetzungen wurde eine Geschäftsordnung des Registers erstellt, die die Registerstruktur und den Datenzugriff regelt, und ein zentrales Ethikvotum bei der Ethikkommission der Friedrich-Alexander-Universität (FAU) Erlangen-Nürnberg eingeholt, an dem sich die externen Zentren orientieren konnten. Die Aufklärung der Patient*innen erfolgte in allen Fällen ausführlich schriftlich vor der jeweiligen Biopsieentnahme. Für jede untersuchte Biopsie wurde ein standardisierter histomorphologischer Befund erstellt. Das Register wurde primär so konzipiert, dass die jeweiligen Einsender nur den Zugriff auf ihre eigenen Daten haben und diese auswerten können. Eine darüber hinaus gehende Auswertung bzw. die Bearbeitung einer speziellen Fragestellung am Gesamtkollektiv ist nur auf Antrag und unter Mithilfe des Administrators möglich ist. Dieses Vorgehen limitiert natürlich die schnelle Verfügbarkeit von Ergebnissen.

Das Konzept dieses klinischen Registers sah vor, bei PD-Patient*innen zu jedem möglichen Zeitpunkt, d. h. im Rahmen von aus klinischer Indikation stattfindenden Eingriffen, Peritonealbiopsien zu entnehmen, diese standardisiert histologisch aufzuarbeiten und die Ergebnisse zusammen mit den relevanten klinischen Parametern in einem Onlineregister zu dokumentieren. Neben Struktur-Funktions-Korrelationen sollten insbesondere auch Verlaufsuntersuchungen einzelner Patienten ermöglicht werden, die dann ggf. die Grundlage für Therapieentscheidungen liefern können. Darüber hinaus sollte der Einfluss bestimmter Therapiemodalitäten, wie z. B. der Zusammensetzung der Dialyseflüssigkeit oder auch der antihypertensiven Medikation, auf strukturelle und funktionelle Veränderungen des Peritoneums untersucht werden. Des Weiteren kann eine standardisierte histopathologische Untersuchung des Peritoneums in Einzelfällen auch über morphologische Zufallsbefunde Erkrankungen diagnostizieren und so die weitere Therapie des Patienten beeinflussen (siehe unten).

## Anatomie und Physiologie des Peritoneums (Abb. 1)

**Figure 1:**
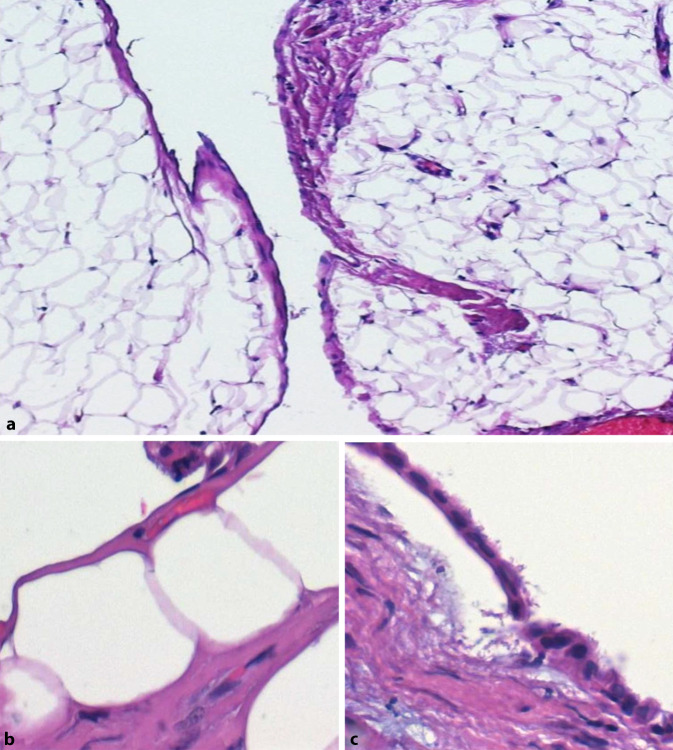

Für die morphologische Analyse der Veränderungen des Peritoneums ist es zunächst notwendig, die normale Anatomie und Physiologie des Peritoneums zu kennen und zu verstehen [1]. Das Peritoneum umgibt mit seinen 2 Blättern (Peritoneum parietale und viscerale) als innere Auskleidung des Bauchraums zahlreiche innere Organe unterhalb des Zwerchfells. Das Peritoneum ist eine seröse Haut, die von einer Mesothelzellschicht (Abb. 1a, b) überkleidet wird. Diese besitzt einen Besatz durch Mikrovilli (Abb. 1c), um die Oberfläche (1,5–2,2 m^2^) zu vergrößern. Zur Tiefe hin folgt dann eine kollagenfaserreiche Bindegewebsschicht, die sog. submesotheliale Kompakta, die eine Dicke von ca. 25–125 µm (Median: 50 µm) aufweist. In dem darunter gelegenen fibrolipomatösen Weichgewebe finden sich dann die Blut- und Lymphgefäße und die Nerven. Das Peritoneum kann Flüssigkeit sezernieren und resorbieren und stellt ein elastisches Aufhängeband für die inneren Organe dar. Die Peritonealflüssigkeit ermöglicht die Verschieblichkeit der inneren Organe, was v. a. für die Funktion von Dünn- und Dickdarm wichtig ist.

## Pathologische Veränderungen des Peritoneums (Abb. 2)

**Figure 2:**
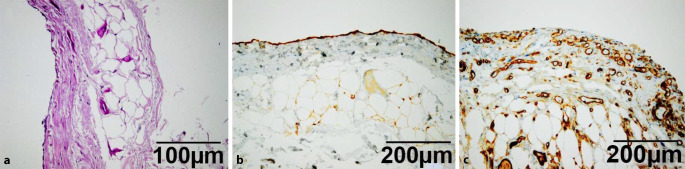

Die pathologischen Veränderungen des Peritoneums [4] entsprechen denen anderer seröser Häute und reichen von akuten und chronischen Entzündungen mit oder ohne Fibrinbelägen bis hin zu einer Fibrosierung der submesothelialen Kompakta (Abb. 2a, b) mit Zunahme der Vaskularisierung, d. h. der Gefäßdichte und Veränderungen der Gefäßwände (Abb. 2c). Art und Ausmaß der morphologischen und funktionellen Veränderungen hängen u. a. auch von den verwendeten Dialysaten ab. So wurde gezeigt, dass sowohl die chronische Niereninsuffizienz selbst [17] als auch deren Therapie mittels PD [18, 19], aber v. a. die Zusammensetzung der für die PD verwendeten Lösungen direkten Einfluss auf die Morphologie des Peritoneums haben [15]. Insbesondere die Fibrose des Peritoneums geht mit einer verminderten Transportkapazität einher und kann schließlich zum peritonealen Versagen, dem sog. Ultrafiltrationsversagen, führen [5]. Laut der verfügbaren Literatur geht man derzeit davon aus, dass es ab einer Dicke der submesothelilaen Kompakta von im Median 350 nm [8] bzw. 750 nm [17] zu einem Ultrafiltrationsversagen des Peritoneums kommt, wobei hier große interindividuelle Unterschiede zu beobachten sind.

**Figure 3:**
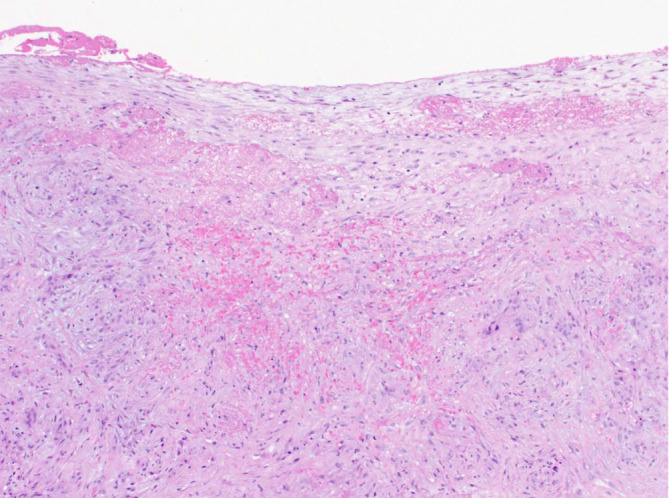

Die schwerwiegendste, zum Glück sehr seltene pathologische Veränderung des Peritoneums stellt die sog. enkapsulierende peritoneale Sklerose (EPS) dar, die mit einer massiven entzündlichen Verdickung des Peritoneums mit einer kokonartigen Ummauerung der Darmschlingen einhergeht und nicht selten eine lebensbedrohliche Komplikation für die Patienten darstellt [3, 7, 10, 11]. Diese gefürchtete Komplikation der PD kommt auch bei pädiatrischen Patienten vor [16]. Charakteristische histologische Veränderungen einer EPS sind neben einer massiven Zunahme der Dicke der submesothelialen Kompakta (in der Regel deutlich über 500 µm) ausgeprägte vaskulopathische Veränderungen sowie oberflächliche Fibrinbeläge und auch Verkalkungen oder sogar Verknöcherungen (Abb. 3; [2, 9, 13, 14]). Jüngere Studien zur Pathogenese der EPS zeigen charakteristische Veränderungen der Expression bestimmter Proteine bei EPS [20], andere weisen auf eine mögliche proinflammatorische Rolle des peritonealen Fettgewebes, speziell der Adipozyten hin [12].

## Chirurgisches Vorgehen bei der Probenentnahme (Abb. 4)

**Figure 4:**
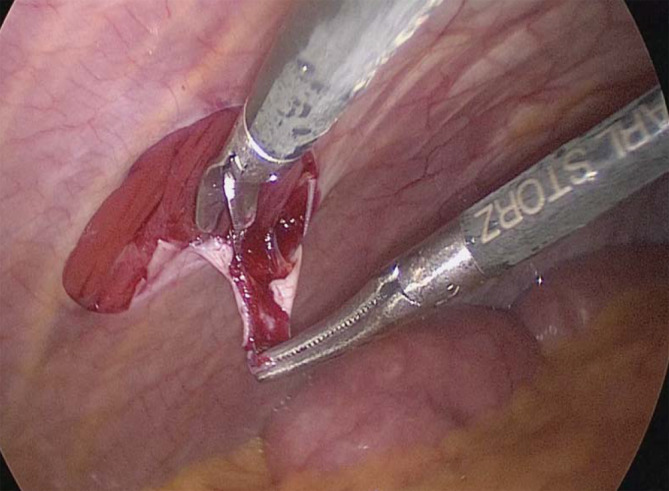

Ziel der Peritonealprobenentnahme (PE) ist es, ein repräsentatives und unbeschädigtes Präparat zu gewinnen, um Artefakte bei der histologischen Untersuchung möglichst zu vermeiden. Dafür ist es besonders wichtig, dass Quetschungen der Probe bei der Entnahme verhindert werden. Ansonsten suggerieren die dadurch entnahmebedingten Befunde nicht real existierende Veränderungen, die von histologischen PD-assoziierten Veränderungen nur schwer differenziert werden können. Zudem muss das Austrocknen der Präparate nach der Entnahme verhindert werden, da auch dies Artefakte bedingen kann. Dies wird u. a. durch atraumatische Entnahmetechniken erreicht, die im Folgenden vorgestellt werden sollen (Abb. 4).

- Allgemeine Aspekte: Es soll eine Probe des parietalen Peritoneums und, falls möglich (z. B. bei größeren abdominellen Eingriffen), zusätzlich auch des viszeralen Peritoneums entnommen werden. Der Abstand der Entnahmestelle vom peritonealen Katheterexit soll mindestens 5 cm betragen. Die Art des entnommenen Peritoneums, der Abstand zum PD-Katheter und die exakte Lokalisation der PE werden in einem standardisierten Formular dokumentiert bzw. skizziert. Das Vorhandensein einer makroskopischen Kokonbildung oder von Belägen (z. B. fibrinös, eitrig) sollten bei der Einsendung angegeben werden.
- Spezielle Techniken: Bei konventionellen Eingriffen mittels (Mini‑)Laparotomie wird eine ca. 1 cm^2^ große Probe des parietalen Peritoneums in sog. Suture-Methode entnommen. Hierfür wird das Peritoneum mittels einer feinen Naht leicht angehoben und von hier aus in o. g. Größe scharf ausgeschnitten. Die Probe wird dann vorübergehend in NaCl‑0,9 %-Lösung gelegt. Bei laparoskopischen Eingriffen wird das parietale Peritoneum mit einer über einen 11-mm-Trokar durch eine Reduzierhülse eingebrachten, laparoskopischen Fasszange vorsichtig angefasst, hiermit ebenfalls angehoben und nicht mehr losgelassen. Sodann wird von hier aus eine etwas mehr als 1 cm^2^ große Fläche mit einer laparoskopischen Schere exzidiert. Die Probe wird nun vorsichtig in die Reduzierhülse zurückgezogen und mit dieser dann herausgezogen. Dann wird die Zange wieder aus der Hülse vorgeschoben, die Probe mit einer Schere von dem gequetschten Anteil scharf abgetrennt und in die NaCl‑0,9 %-Lösung eingelegt. Somit wird sichergestellt, dass an der eingesandten Probe kein Entnahmetrauma vorliegt. Im Kölner Zentrum wird die Probe regelhaft vom parietalen Peritoneum im rechten Oberbauch in Höhe des Rippenbogens entnommen, sodass die vorgeschriebene Mindestentfernung zum Katheter eingehalten wird. Eine eventuell notwendige Blutstillung an der Entnahmestelle erfolgt erst nach Abgabe der Probe.

**Figure 5:**
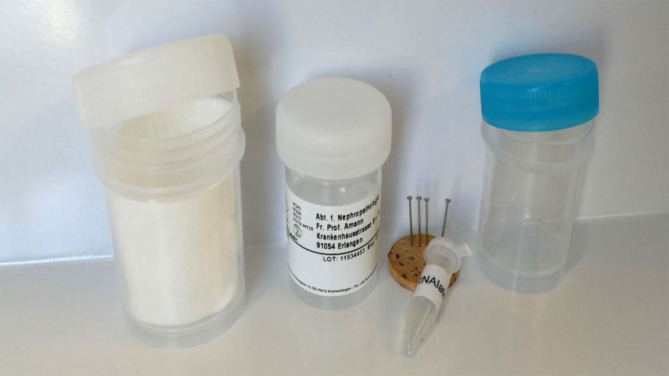

Nach Eingriffsende wird die Probe mit einer Schere halbiert und die eine Hälfte in ein mit RNAlater gefülltes, 1,5 ml fassendes Röhrchen gegeben, die andere Hälfte auf einem Korkplättchen aufgespannt (Serosaseite nach oben weisend) sowie in gepuffertes Formalin gegeben, wobei die Korpusrückseite mit dem Gewebe nach unten zur Flüssigkeit zeigt (Abb. 5). Die beiden Gefäße werden mit dem ausgefüllten Formular zusammen in das pathologische Referenzzentrum eingesendet.

Klinische Angaben, die in das Register einfließen:

Nach Durchsicht der einschlägigen Literatur und in Absprache mit den einsendenden Zentren wurden die folgenden *klinischen Angaben* für das Register definiert:

1. Patientendaten: behandelndes Zentrum, Patienten-ID, Geburtsdatum und Geschlecht.
2. Therapiedaten: Dauer der PD-Behandlung, Angaben zum Dialysat, Dauer der Nierenersatztherapie insgesamt (PD und HD), PD-Regime, Transportertyp (nach PET-Test), ACE-Hemmer-Therapie (ja/nein), Diabetes (ja/nein), Nikotinabusus (ja/nein), Restnierenfunktion (Diurese und Kreatinin-Clearance), nephrologische Grunderkrankung.
3. Peritonealbiopsie: Katheterimplantation, Katheterexplantation, Peritonitis, Ort der Entnahme (viszeral, parietal, Omentum). Bei parietalem Peritoneum Abstand zum PD-Katheter in cm (bei PD-Katheter-Anlage oder -Entfernung parietales Peritoneum, bei EPS immer zusätzlich viszerales Peritoneum), Entnahmetechnik (suture vs. konventionell). Schaubild: Abdomen, Katheterlage, Ort der Biopsieentnahme, Konservierung des Peritonealgewebes in Formalin, Flüssigstickstoff, RNAlater, sonstige.

## Histomorphologische Aufarbeitung von Peritonealbiopsien (Abb. 5 und 6)

Neben den o. g. Entnahmevoraussetzungen stellt die orthograde Ausrichtung der Peritonealbiopsieproben bei der Einbettung eine weitere wichtige Voraussetzung für alle qualitativen und quantitativen morphologischen Untersuchungen des Peritoneums dar. Dies wird unterstützt durch eine spezielle Logistik, die den einsendenden Zentren Korkplättchen und Nadeln für die Fixation der Peritonealbiopsieproben zu Verfügung stellt (Abb. 5). Durch entsprechende Tuschemarkierungen wird beim Zuschnitt und der Einbettung der aufgespannten formalinfixierten Proben sichergestellt, dass eine orthograde Ausrichtung im Schnittpräparat gewährleistet ist. Dies gelingt in der Regel nur zuverlässig ab einer bestimmten Mindestgröße der Biopsie. Die in RNAlater eingesandte Probe wird für ergänzende molekulare Untersuchungen asserviert und kann im Rahmen von Forschungsprojekten auf Antrag für bestimmte Fragestellungen zur Gen- oder Proteinexpression genutzt werden. Die formalinfixierten Proben werden gekantet in Paraffin eingebettet und es werden jeweils eine HE-, PAS- und eine Siriusrot-Färbung angefertigt. Darüber hinaus werden routinemäßig jeweils eine Calretinin- und eine CD31-Immunhistologie zur Darstellung der mesothelialen Überkleidung bzw. der Blutgefäße angefertigt. Die Messung der Dicke der submesothelialen Kompakta erfolgt an den HE-Schnitten durch Erhebung der minimalen und maximalen Werte mittels eines halbautomatischen Bildanalysesystems und Angabe der sog. „Range“ der Messwerte.

**Figure 6:**
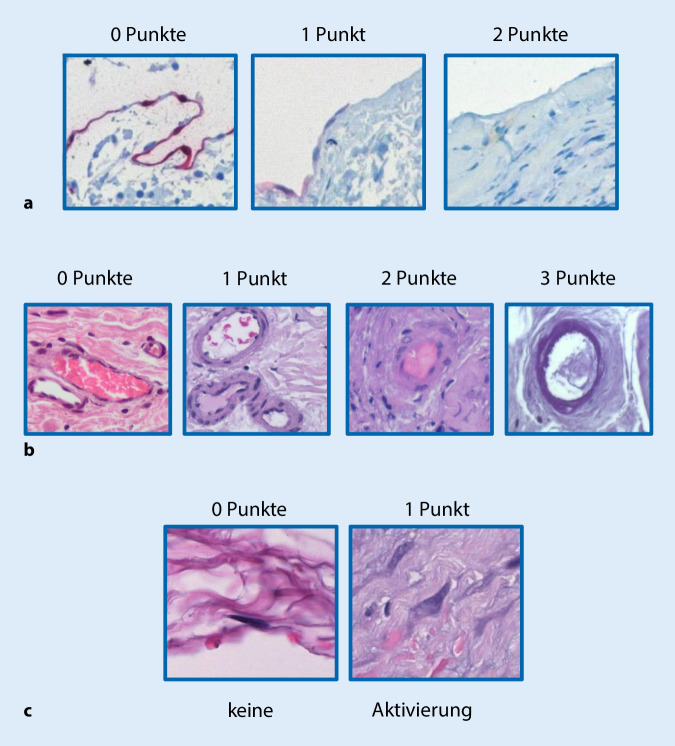

**Figure 7:**
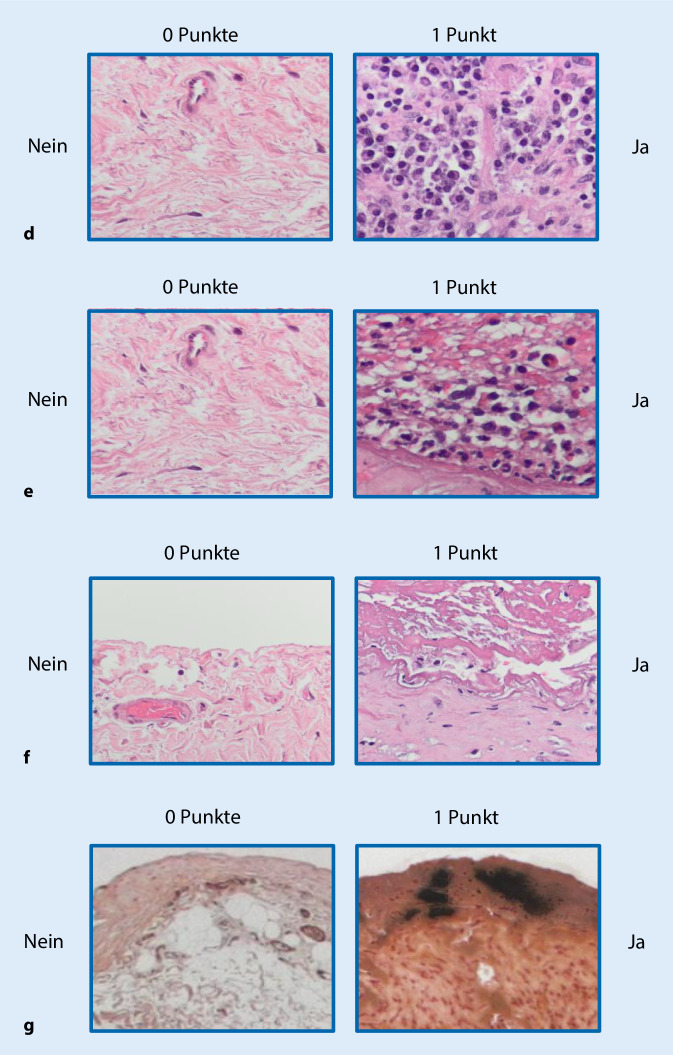

Zur Erfassung der strukturellen Veränderungen des Peritoneums wurden im Vorfeld auch anhand der in der Literatur verfügbaren Daten die folgenden histologischen Parameter identifiziert, die entweder mithilfe semiquantitativer Scores (0–3) (Abb. 6a, b) bzw. nach Fehlen oder Vorhandensein (Abb. 6c–g) beurteilt wurden: a) Integrität der mesothelialen Deckzellschicht, b) Vaskulopathie, c) Fibroblastenaktivierung, d, e) chronische und floride Entzündung, f) Fibrinabscheidungen und g) Kalzifikationen oder Ossifikationen. Aus der Addition der Einzelscores kann ein Gesamtscore der morphologische Veränderungen ermittelt werden, der v. a. auch hilfreich für korrelative Analysen, z. B. zur Struktur-Funktions-Beziehung, sein soll. Bislang wurden auf diese Weise insgesamt 1692 Peritonealbiopsien morphologisch untersucht und die Ergebnisse zusammen mit den klinischen Parametern im Register dokumentiert (Abb. 7). Aus logistischen Gründen liegen bislang nur wenige register- oder zentrumsspezifische Ergebnisse vor. Mithilfe des o. g. Gesamtscores konnten wir jedoch, z. B. an einer Subgruppe, zeigen, dass die Dicke der submesothelialen Kompakta und die gesteigerte Neoangiogenese jeweils sehr gut mit dem Vorhandensein einer floriden und chronischen Entzündung korrelieren. Weiterhin fanden wir, dass viele Patienten über Jahre hinweg sehr stabile Messwerte der submesothelialen Kompakta und auch einen niedrigen histologischen Gesamtscore zeigen, während es wenige Patienten gibt, die schon nach kurzer Zeit deutliche Anstiege der submesothelialen Kompakta und des Gesamtscores aufweisen und dann in der Regel einen schlechten Verlauf zeigen. In einer weiteren Subanalyse ergaben sich darüber hinaus beispielsweise auch interessante Korrelationen zu den renalen Grunderkrankungen der Patienten. Weitere Subanalysen des größten einsendenden Zentrums zeigten keinen Zusammenhang zwischen der Kompaktadicke und den verwendeten PD-Verfahren. Weiterhin konnten wir in unserem Register den in der Literatur postulierten Zusammenhang zwischen Diabetes mellitus und Zunahme der Peritonealdicke bislang nicht bestätigen.

**Figure 8:**
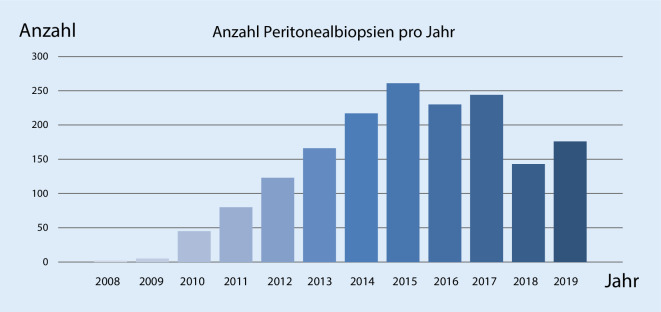

## Ausgewählte histomorphologische Befunde des Peritoneums bei PD (Abb. 8 und 9)

Die standardisierte histologische Untersuchung des Peritoneums ergab zum einen interessante Einzelfallverläufen, die trotz eines vergleichbaren Behandlungsregimes einen individuell völlig unterschiedlichen Verlauf der peritonealen Veränderungen dokumentieren (Abb. 8). Ein Therapieversagen aufgrund von Strukturveränderungen zeigt sich nach klinischen Erfahrungen frühestens nach 3–5 Jahren unter PD. Die Verdickung des Peritoneums auf das 6‑ bis 8‑fache ist bei dem vorliegenden Patienten im Verlauf von 1,5 Jahren sehr ungewöhnlich und wäre ohne die initiale Biopsie bei Katheteranlage nicht feststellbar gewesen (Verlauf). In diesem Fall führte der V.a. eine Katheterfehlfunktion zu einer laparoskopischen Revision des Katheters. Dabei wurde die Verlaufsbiopsie entnommen. Das histologische Ergebnis zeigte eindeutig, dass hier die Strukturveränderung des Peritoneums für den Funktionsverlust verantwortlich war. Daher erfolgte eine Umstellung des Verfahrens auf Hämodialyse (HD). Ohne die vorliegende Histologie des Peritoneums wäre hier sicher ein weiterer Therapieversuch mit PD erfolgt.

**Figure 9:**
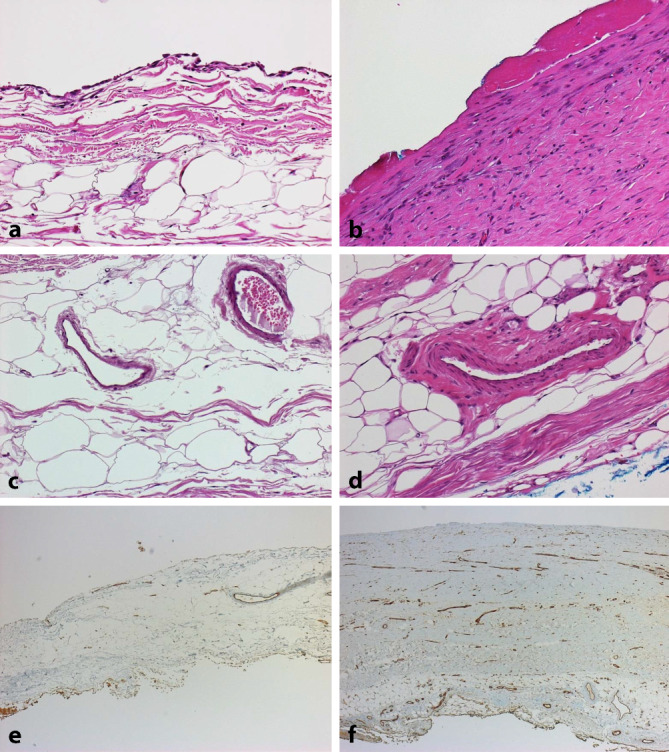

**Figure 10:**
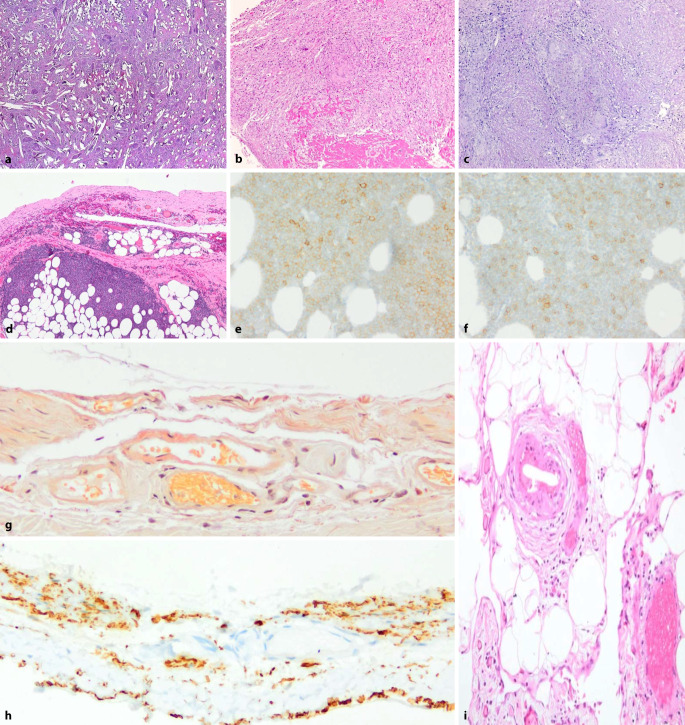

Zum Anderen ergaben sich auch eine Reihe unerwarteter und klinisch höchst relevanter morphologischer Befunde (Abb. 9). So wurden in den Peritonealbiopsaten als Zufallsbefunde beispielsweise neben den häufigen Fremdkörper‑/Fadengranulomen als Residuen der Katheterimplantation (Abb. 9a) in jeweils einem Fall auch eine nekrotisierende epitheloidzellig-granulomatöse Entzündung im Sinne einer Peritonealtuberkulose (Abb. 9b, c), eine Infiltration des Peritoneums bei bis dahin unbekannter chronisch-lymphozytischer Leukämie der B‑Zell-Reihe (B-CLL; Abb. 9d–f), eine Mitbeteiligung des Peritoneums bei AA-Amyloidose (Abb. 9g, h) sowie eine Cholesterinkristallembolisation (Abb. 9i) als überraschende Ursache des Funktionsversagens des Peritoneums gefunden. In einem Fall wurden auch Infiltrate eines bislang unbekannten Karzinoms im Peritonealbiopsat diagnostiziert.

## Schlussfolgerungen und Fazit für die Praxis

Das Deutsche Peritonealbiopsieregister (GRIP) hat sich als eine erfolgreiche und wichtige Struktur in der deutschen Nephrologie etabliert. Es liefert klinisch relevante Informationen über pathologische Veränderungen des Peritoneums und stellt darüber hinaus wertvolles Biopsiematerial für klinische und wissenschaftliche Fragestellungen zur Verfügung. Des Weiteren erlauben die in den Peritonealbiopsien erhobenen Zufallsbefunde eine frühzeitige Diagnostik und Behandlung bestimmter Erkrankungen. Die Auswertungen der morphologischen und funktionellen Veränderungen des Peritoneums sind am Gesamtkollektiv oder zentrumsbezogen möglich und versprechen interessante Einsichten und wertvolle Daten, die zu einem besseren Verständnis der Umbauvorgänge des Peritoneums und möglicherweise auch einer veränderten therapeutischen Vorgehensweise beitragen können.

Die Kenntnis der normalen Struktur des Peritoneums und seiner pathologischen Veränderungen ist für Pathologen allgemein interessant und wichtig, da neben den PD-assoziierten Veränderungen auch zahlreiche Manifestationen anderer Erkrankungen am Peritoneum diagnostiziert werden können.
